# “Infinitesimalism Versus Science”

**Published:** 1883-10

**Authors:** H. M. Corey


### "INFINITESIMALISM VS. SCIENCE."

H. M. COREY, M. D.

In the July number of THE BISTOURY there appeared an article in which the writer hurls a scientific shaft straight at the vitals of homœopathy, especially the "high dilutionists;" and we are informed by an editorial in the same number that it struck a vulnerable point and must prove fatal—that is, if the "new chemistry" is correct. Now, Mr. Editor, the "new chemistry" is undoubtedly correct, so far as it is concerned in the article named, and the quotation marks will enable us to make no mistake in the amount to which it is involved, viz: "Equal volumes of all substances, when in a state of gas and under like conditions, contain the same number of molecules."

Now, this is the sum total of the new chemistry engaged in this terrible combat, in which "Science" attempts to hunt down and throttle the poor, little, strayed molecule, after graphically describing the manner in which the poor little waif becomes separated from its companions in their subjection to the homœopathic methods of dilution.

So far, then, "Science" has shown that there are molecules—aconite for example—staggering around amongst a myriad of alcohol molecules.

There has not been quite enough science brought to bear yet, to determine the exact number; but, backed up by the "new chemistry," that is about to be done. And, now, let us see how it is accomplished.

1st. A very simple arithmetical statement is submitted to show that in the second centesimal dilution of any drug, the molecules composing it are separated and mixed with those of alcohol, through a space ten thousand times as great as they originally occupied.

2d. A well-known philosophical law, that the molecules of water, while in a state of steam, are separated and diffused through a space only seventeen hundred times as great as they occupied as a liquid. Both of which are undoubtedly correct.

Now, it appears that this is as far as "Science" can go unaided, so, slipping the collar from the "new chemistry," by quoting [as we are informed] two of its underlying laws, puts it upon the scent, and soon assures us that the game is fairly brought to bay. The first of these laws we quoted in the beginning of our article. The second is not inclosed in quotation marks; hence we infer that that underlies the chemistry that is yet to be written.

Now, for convenience, we will quote both laws together, as "Science" does, for a starting point:

1st. "Equal volumes of all substances, *when in a state of gas and under like conditions*, contain the same number of molecules."

2d. "The number of molecules in a cubic inch of *gas, at a temperature of* 32° *Fahrenheit*, with an atmospheric pressure as indicated by the barometer at 30 inches, is 10 or $10^{23}$, raised to the twenty-third power, which is 1,000,000,000,000,000,000,000,000, or one septillion." [The italics are ours.]

Now, if these laws mean anything, they mean just what they say; and it would seem superfluous for us to add, in connection with them, that they relate to gases and gases only, and permanent gases at that—not vapors. And yet, right here, is where the concentrated quintessence of all the sciences combined come in to catch the molecule. Now watch! "Now suppose," Science continues, "that we have before us a bottle holding a cubic inch of the second dilution of aconite, and another a cubic inch of steam. In the case of steam we have one septillion molecules, as we have shown, while the interstices between the molecules have been widened only seventeen hundred times; while in the case of aconite molecules we have widened the interstices ten thousand times."

Now, I ask, in all candor, can it be possible that the writer means that?—means it to be a scientific deduction from the laws quoted, and held up as his guide? It must be so, for farther on he says, very loudly, that he "challenges refutation," and it will be further seen his whole calculations are based upon the supposition that he has proven that a cubic inch of steam contains a septillion of molecules, no more and no less, but why he thinks so, is more than I can, from this article, learn. Certainly, his "new chemistry" teaches no such thing, and there is but one thing it can teach. and that is this: That a cubic inch of steam, subjected to the conditions demanded by his second law, viz., reduced to a temperature of 32° Fahrenheit, under an atmospheric pressure of 30 inches, would be no longer a gas, or even a fluid, but, allowing for the slight expansion that water undergoes during congealation, he would have a little over $\frac{1}{1700}$ part of a cubic inch of ice. Surely that throws an immense amount of light upon the number of molecules it contains.

Yet, I think it does show, quite conclusively, that the interstices *are not* widened 1,700 times, and would afford any but a scientist but poor proof that the ultimate limit to the divisibility of matter is reached at any particular step in the homœopathic list of dilution, be it 13th or 30th, and we leave it for the reader to judge in which case the greater height of absurdity is reached: In accepting conclusions, verified time after time, by experience, or to be guided by—by—"Science."

